# The National Italian Guidelines on the diagnosis and treatment of children with pediatric ataxias

**DOI:** 10.3389/fneur.2022.971781

**Published:** 2022-10-04

**Authors:** Eleonora Lacorte, Paola Piscopo, Luciano Sagliocca, Luca Vignatelli, Domenica Taruscio, Nicola Vanacore, The Ataxia Guideline Development Working Group (GDWG), I Bacigalupo

**Affiliations:** ^1^Centro Nazionale per la Prevenzione delle Malattie e la Promozione della Salute, Istituto Superiore di Sanità, Rome, Italy; ^2^Dipartimento di Neuroscienze, Istituto Superiore di Sanità, Rome, Italy; ^3^Azienda Sanitaria Locale, Salerno, Italy; ^4^IRCCS Istituto delle Scienze Neurologiche di Bologna, Bologna, Italy; ^5^National Center for Rare Diseases, Italian National Institute of Health, Rome, Italy

**Keywords:** guidelines, recommendations, pediatric ataxias, diagnosis, treatment, rare diseases

## Abstract

**Background:**

Ataxia is a rare neurological condition causing a deficit in the coordination of motor activities, preventing the fluidity of movements. Children with ataxia may show several different ataxic signs, along with difficulties in walking autonomously and ataxic gait often associated with trunk instability. Ataxic signs can be either acute or chronic, and in either case, the diagnosis can be extremely complex. Symptoms and their etiology are often widely heterogeneous, even within the same condition.

**Methods:**

The guideline was developed based on the methodology defined by the Methodological Handbook of the Italian National Guideline System (SNLG) and was reported following the AGREE-II checklist. The SNLG methodology required the adoption of the GRADE approach for the whole development process. To facilitate the implementation of the contents and recommendations from the guideline, two care pathways were developed based on the NICE and the European Pathway Association (EPA) models.

**Results:**

The guideline included 28 clinical questions, 4 on the identification and management of acute ataxias, and 24 on the diagnosis and management of chronic ataxias. The document included 44 recommendations, 37 clinical recommendations, and 7 recommendations for research.

**Conclusion:**

The working group, despite the lack and methodological limitations of the evidence, deemed as essential to provide indications and recommendations, in particular in some clinically relevant areas. The care pathway was produced as a tool to facilitate the implementation of the contents and recommendations. The interactive version of the pathway is available on the SNLG website along with a leaflet dedicated to families and caregivers.

## Introduction

Ataxia is a rare neurological condition causing a deficit in the coordination of motor activities, preventing the fluidity of movements. Children with ataxia may show several different ataxic signs, including appendicular ataxia, tremor, dysarthria, and oculomotor apraxia, along with difficulties in walking autonomously and ataxic gait often associated with trunk instability. Ataxic signs can be either acute or chronic, and in either case, the diagnosis can be extremely complex. Symptoms and their etiology are often widely heterogeneous, even within the same condition.

Ataxias are anatomically classified as cerebellar, cerebral, sensory, and labyrinthine ataxias. Acute cerebellar ataxias are the most frequent form of acute ataxia in children, with an estimated incidence of 1/100,000 children. Acute ataxias are mostly benign, but acute ataxic signs can also be due to severe conditions that can be life-threatening. A timely diagnosis is therefore essential to define its cause (1–5).

Chronic ataxias are, instead, typically due to genetic mutations. These conditions can be progressive or non-progressive and can be caused by neuro-pathological anomalies of the cerebellum, mainly of the degenerative type, with some cases of the malformation type (e.g., hypoplasia or dysplasia) that may be associated with a neurodegenerative component. The prevalence of chronic ataxias in Europe is estimated to be 26/100,000 children aged 0–19 years (1, 6).

Several types of ataxias do not show any specific neuroimaging sign, thus making their diagnostic process extremely complex. Moreover, the genetic diagnosis can be affected by the limited availability of tests (the genetic centers that carry out these tests are few), the wide heterogeneity and non-standardization of types of tests (each center uses its own panel of genes) and their use in the centers offering genetic testing, and by the limited knowledge on the genetic basis of some of the types of ataxias. This can cause a diagnostic delay but can also affect the prognosis and the counseling process. Reaching a timely diagnosis is essential to ensure that children can access early treatments. Though currently, no disease-modifying treatments are available for most of the chronic ataxias, several aspects of these conditions can be treated with timely interventions. Therefore, timely and appropriate identification and diagnosis are essential. In particular, motor and cognitive rehabilitation are crucial to improve the quality of life and support the inclusion of children in their social environment. For this reason, rehabilitation plans should not only be accessible in specialized centers but they should also be offered by territorial services through a coordinated care approach between the referral centers and the territorial services (1, 7–9).

To our knowledge, no updated national guidelines have been developed through a standardized and shared methodology by governmental agencies at an international level. Therefore, this is the first guideline produced based on an internationally shared, standardize, and rigorous methodology covering the diagnosis and management of childhood ataxias.

This guideline is addressed to all healthcare and social care professionals involved in the diagnosis and management of children with ataxia, to all decision-makers involved in organizing care for these patients, and to all other stakeholders, such as families and caregivers, to provide them with indications for best practices for the diagnosis and management of children with ataxia. It has also the objective to provide tools for a more efficient organization of the care process and for supporting research in this field. As a support to facilitate the dissemination and implementation of the recommendations and contents of the guideline, a care pathway has also been developed, with the objective of providing a practical visual tool for organizing the care process.

## Methods

The guideline was developed following the methodology defined by the Methodological Handbook published by the National Guideline System (Sistema Nazionale Linee Guida—SNLG) of the Italian National Institute of Health (Istituto Superiore di Sanità—ISS) (10) and was reported according to the AGREE-II checklist (11). The SNLG methodology required the adoption of the GRADE approach (12) for the whole development process. A multidisciplinary panel was nominated through a public procedure. It included representatives of all the healthcare professionals involved in the diagnosis and management of children with ataxias (including 2 child and adolescent psychiatrists, 1 engineer, 1 geneticist, 2 neuro-geneticist, 2 neurologists, 1 occupational therapist, 2 pediatricians, 2 psychologists, 1 physiatrist, 3 physiotherapists, and 2 speech and language therapists), and representatives of family members and caregivers. All members of the Guideline Development Group (GDG) and the multidisciplinary panel signed a standardized form for declaring any competing and conflicts of interest, and participated in the GL as individual entities, and not as representatives of specific organizations, associations, or scientific societies. All forms are available as supplementary material on the SNLG website. The scope and clinical questions were drafted and approved by the panel, and systematic reviews were conducted for each clinical question. All available evidence was retrieved through specific searches produced by specifically trained documentalists. Searches were kept as wide as possible to avoid missing relevant studies, and, where necessary, indirect evidence was also considered. Evidence was qualitatively assessed and summarized in evidence tables, and recommendations were defined based on results from the evidence review and discussion with the panel. In fact, some recommendations were produced even in absence of evidence, but only in strictly specific cases in which the recommended indication was the only reasonable and acceptable behavior (e.g., admission in ED in case of children with acute, life-threatening signs of ataxia). In case of missing or extremely scarce evidence, no recommendations were issued, except for research recommendations in case of issues requiring further research. Recommendations were graded according to the GRADE method as either weak or strong based on the evidence to decision framework (i.e., considering the relevance of the issue, benefit/risk tradeoff, equity, acceptability, feasibility, and use of resources). All recommendations and clinical indications produced within this guideline were aimed at reducing the variability and inappropriateness of clinical behaviors in the management of children with ataxia within the Italian territory. The main duty of a public-funded, nationally endorsed guideline is to increase the efficiency and appropriateness of clinical behaviors to ensure that the best healthcare is provided to the patients, while guaranteeing that the provided indications are feasible, applicable, and implementable at a national and local level, to avoid producing theoretical documents that might not produce effective improvements in clinical practice. Research recommendations were also produced to support future research on areas where evidence was scarce or lacking.

Being ataxias a group of rare diseases, GDG adopted, as a further methodological source, specific indications for the application of the GRADE approach in guidelines for rare diseases, provided by the RARE-Best practices Consortium (13). Based on these indications, the main issues in the application of the GRADE approach regarding rare diseases concerned the systematic review process. When considering search strategies, the GRADE approach requires the definition of a search string for each considered review question. However, literature on rare diseases can be extremely scarce and heterogeneous, and sometimes its identification might be limited by indexing issues. Therefore, the GWD adopted a more sensitive and less specific approach when structuring search strategies, by building search strings for topic areas (e.g., diagnosis and pharmacological treatment) to maximize the chances of retrieving all potentially relevant literature (13). The scarcity and heterogeneity of literature was also the reason leading the GWR to adopt both tabular and narrative approaches to the summary of evidence rather than only a strictly tabular one. The option, provided by the GRADE Handbook, was adopted to allow for a more in-depth contextualization of evidence and the possibility of discussing its relevance despite its potential methodological limitations (12). Carrying out high-quality studies on rare diseases can be extremely challenging, due to several obstacles including the difficulties of enrolling large and/or homogeneous samples, and providing standardized treatments (13–15). This is most evident in ataxias, as the clinical and phenotypic presentation of disease can be very heterogeneous even among the same genotypes. This variability in signs and symptoms and their severity leads to the need of providing tailored interventions targeted to individual needs and outcomes. To contextualize the evidence to each phase of the care process and to account for this variability, outcomes were therefore considered in a more transversal way, as they could have a different weight according to each clinical situation. Recommendations were also provided for each area, as the main objective of the GWD and the panel was to provide a clear indication and a clear representation of the whole care process, from the identification and diagnosis to the management and definition of therapeutic strategies. For this purpose, to further underline the relevance of providing an indication of flow of care and to facilitate the implementation of the contents and recommendation from the guideline, two care pathways were developed based on the NICE (16) and the European Pathway Association (EPA) models (17). The first pathway included all recommendations on acute ataxias, and the second pathway included all recommendations on chronic ataxias, including a sub-pathway for genetic testing. Based on the definition provided by the European Pathway Association, we included in the development of this pathway all the key elements considered useful to offer patients and their caregivers evidence-based care.

The final draft of the GL defined by the GdL and the multidisciplinary panel was uploaded to the SNLG website for open review. After the open review phase, the document was adjusted for the received comments and was sent to two independent reviewers for a peer review. Comments from the peer reviewers were discussed with the panel, and the document was revised accordingly. Before publication, the document underwent the last review process by two reviewers from the SNLG, including a structured assessment using the AGREE tool.

Considering the constantly and rapidly growing research and knowledge in this field, the update of this GL was set in January 2024.

The production of the GL was one of the specific objectives included in a national project funded by the Italian Ministry of Health: “NET-2013-02356160—Pediatric ataxias and Public Health: epidemiological studies and disease registry, characterization of genetic determinants and implementation of protocols for diagnosis, management, and rehabilitation using innovative low cost, widely accessible technologies.” The funder body was not involved in the production of the GL, thus its positions, views, and opinions did not influence the final recommendations and contents of the GL.

## Results

The guideline included 28 clinical questions, which were divided into two different sections. The first section included four clinical questions on the identification and management of acute ataxias, while the second section included 24 clinical questions on the diagnostic process, management, care organization, and care models for chronic ataxias. The document included 44 recommendations, of which 37 clinical recommendations (Table 1) and 7 recommendations for research (Table 2). Recommendations were graded as either strong or weak, with 26 recommendations graded as strong, and 11 graded as weak.

**Table 1:** List of the recommendations included in the guideline classified according to the areas identified in the care pathway for both acute ataxias **(A)** and chronic ataxias **(B)**.

**(A)**		
Onset	Children with signs of acute ataxia should be referred to or transferred to a specialized pediatric structure only after being stabilized (ERC Guidelines for resuscitation 2015).	Strong recommendation
Visit by a specialist	Children referred to an ER for acute ataxia should undergo an accurate anamnesis and a general and specialized objective examination aimed at identifying the possible causes of the signs and symptoms, and at choosing of the type and order of instrumental examination to be carried out.	Strong recommendation
Toxicological screening	Children with signs of acute ataxia whose anamnesis and/or test results do not univocally suggest a defined etiology should be further assessed by performing toxicological screening (e.g., alcohol, amphetamines, cannabinoids, benzodiazepines, ecstasy, methadone, cocaine, opioids).	Strong recommendation
CSF test	Children with acute ataxia and a suspected infection of the central nervous system (e.g., meningitis, encephalitis) should undergo CSF examination if having no contraindications to lumbar puncture*.	Strong recommendation
EEG	Children with a suspected epileptic seizure, altered state of consciousness or fluctuating signs should undergo EEG or video EEG.	Strong recommendation
MR and/or CT	Children showing persistent or isolated signs of acute ataxia lasting for more than 3 days, children showing focal signs, visible asymmetric ataxic signs, altered state of consciousness, cranial neuropathy, papilledema and ophthalmoplegia, children with a suspected demyelinating or vascular disease, and children with an anamnesis of head trauma should undergo brain CT and/or MR.	Strong recommendation
MR and/or CT plus other examinations	Children with suspected paraneoplastic ataxia and children with suspected opsoclonus myoclonus syndrome should undergo brain MR or CT, along with chest RX, abdominal, pelvic and neck ultrasonography, and should be tested for urinary catecholamine metabolites to exclude potential occult neuroblastomas.	Strong recommendation
**(B)**		
Specialized referral center	A coordinated care approach can be useful in the management of children with chronic ataxias and their family members/caregivers, by creating networks of specialists throughout the territory, which should be coordinated by the specialized referral center acting as the access point.	Weak recommendation
Magnetic resonance (MR)	All children with suspected chronic ataxias should undergo at least one brain MR, that should be performed in the early phase of the diagnostic workup by a center with specific expertise in the diagnosis of pediatric ataxias, as the presence of specific morphological characteristics can help defining the type of genetic test to be performed and, in some cases, support the differential diagnosis among the different causes of ataxia (see Annex A in Table 3).	Strong recommendation
Biochemical examinations (diagnostic process)	Observing low levels of CoQ10 in the muscle tissues of children with a diagnosis of chronic ataxia can be suggestive of potentially treatable causes of ataxia due to a deficit of CoQ10. Observing high levels of AFP in children with suspected chronic ataxia, along with a profile of low levels of IgA, IgE and IgG and a low lymphocyte count (CD4+ and CD8+), is suggestive of and supports an early diagnosis of Ataxia telangiectasia.	Weak recommendation Strong recommendation
Electromyography (EMG)/Nerve conduction study (NCS) (diagnostic process)	Children with chronic ataxia, particularly at a very young age, whose test results do not univocally suggest a defined diagnosis, can be further assessed by performing an EMG and/or a nerve conduction study (NCS) (peripheral study).	Weak recommendation
Clinical examination	Children with a diagnosis of chronic ataxia should undergo a global clinical assessment aimed at identifying possible malformations and/or deformities (e.g., pes cavus, scoliosis) and, in case, assessing their severity and progression.	Strong recommendation
Biochemical examinations (clinical/functional assessment)	Monitoring Ig levels (IgA, IgE, IgG, IgM) and lymphocyte count in children with ataxia telangiectasia, can be useful to characterize an immunodeficiency profile and therefore prevent the risk of infections. Observing high levels of IgM in children with a suspect of ataxia telangiectasia is suggestive of a phenotype characterized by a higher risk of infection and earlier mortality.	Weak recommendation Strong recommendation
Liver function tests (clinical/functional assessment)	Children with ataxia telangiectasia, starting from their tenth year of age, could benefit from a liver function test aimed at identifying possible liver dysfunctions and, in case, their severity and progression.	Weak recommendation
Electrocardiography (ECG), echocardiography (clinical/functional assessment)	Children with Friedreich ataxia, even if having no signs of cardiomyopathy, should undergo a clinical, electrocardiographic, and echocardiographic exam aimed at identifying possible cardiac anomalies (e.g., hypertrophic cardiomyopathy) and, in case, assessing their severity and progression.	Strong recommendation
Radiographic (RX) examinations (clinical/functional assessment)	Children with chronic ataxia should undergo a specific radiographic examination aimed at identifying the presence of scoliosis and, in case, assess its progression. Children with ataxia telangiectasia who have scoliosis should not undergo repeated radiographic examinations to assess the progression of scoliosis.	Strong recommendation Strong recommendation
Ophthalmic examination (clinical/functional assessment)	Children with chronic ataxia should undergo an ophthalmic and visual examination aimed at identifying possible neuro-ophthalmic and visual symptoms.	Strong recommendation
Audiometric examination (clinical/functional assessment)	Children with Friedreich ataxia could benefit from undergoing a clinical and audiometric examination aimed at identifying possible hearing deficits and, in case, assessing their severity and progression.	Weak recommendation
Magnetic resonance (MR) imaging (clinical/functional assessment)	Children with a diagnosis of chronic ataxia should not undergo repeated brain MR to assess the progression of ataxic signs and symptoms.	Strong recommendation
Counseling	Family members/caregivers of children with a diagnosis of ataxia should be offered psychological, psychosocial and/or psychoeducational support aimed both at facilitating the access and orientation within healthcare services, and at creating a care coordination process minimizing the burden for family members/caregivers. A coordinated care approach can be useful in the management of children with chronic ataxias and their family members/caregivers, by creating networks of specialists throughout the territory, which should be coordinated by the specialized referral center acting as the access point. Family members/caregivers should be involved in all the phases of the diagnostic and care process of children with chronic ataxias.	Strong recommendation Weak recommendation
Pharmacological treatments	In children with Friedreich ataxia, the treatment with idebenone appears to be not effective in reducing ataxic signs. In children with Friedreich ataxia, the treatment with interferon gamma-1b appears to be not effective in reducing ataxic signs. A specific treatment with vitamin E or CoQ10 supplements should be administered in children with a genetic mutation responsible for a deficit of vitamin E or of CoQ10.	Weak recommendation Weak recommendation Strong recommendation
Habilitative/rehabilitative treatments	Children with a diagnosis of chronic ataxia should be offered a motor and cognitive multi-dimensional neuro-rehabilitative treatment in association with orthoses or aid, when needed, aimed at reaching specific objectives, including self-determination, defined within a shared rehabilitative project taking into consideration the abilities, competences, and performances of each single child, of the type and severity of the specific ataxia, and of the functional prognosis, when known. The rehabilitative project of children with ataxia should be based on a preliminary multi-professional assessment of the abilities, competences, and performances of each child, of the type and severity of the signs and symptoms, and of the functional prognosis, and the coordinated intervention of all professionals should be defined based on the results of such assessment. The use of exergames-based interventions, including the home-based interventions, for the rehabilitation children with chronic ataxia appears to be useful in reducing motor sign and symptoms and in improving balance.	Strong recommendation Strong recommendation Weak recommendation
Pharmacological treatments for associated symptoms	In children with a diagnosis of chronic ataxia who have associated signs and symptoms, these sign and symptom should be treated as for the standard treatment recommended for each of them, taking into consideration the clinical situation of each child, and except in case the administration of the selected treatment could cause a risk and/or damage higher than the expected benefit.	Strong recommendation
Habilitative/rehabilitative treatments for associated symptoms	Specific rehabilitative strategies should be adopted for the treatment of potential sign and symptoms associated to each type of ataxia, taking into consideration the clinical situation of each single child, and except in case the administration of the treatment might cause a risk and/or damage higher than the expected benefit. Specific orthoses and aids should be adopted in children with chronic ataxia who have malformations and/or deformities and/or other clinical conditions requiring them, except in case the adopted orthosis or aid is not well tolerated or causes children a limitation in movements, gait and/or activities of daily living.	Strong recommendation Strong recommendation
**Genetic testing**		
Genetic testing	Children with chronic ataxia should be offered genetic testing to define and confirm the diagnosis. The test should be prescribed by a clinical geneticist following an accurate clinical examination aimed at defining the type test(s) and, in case of multiple tests, their sequence. After being prescribed, the test(s) should be performed only by specialized centers and within a structured path, possibly coordinated by a multidisciplinary team, which should include genetic counseling (e.g., addressing reproductive choices).	Strong recommendation
Genetic counseling	Children with chronic ataxia and their family members/caregivers should be offered genetic counseling interventions that should be coordinated by clinical geneticists or neurologists with specific expertise in the genetics of ataxias, and should include psychological support to the patients, assure their independence from the counselor in making decision, take into consideration the implication of testing family members and of possible reproductive choices, and provide children or their family members/caregivers a written report of the counseling.	Strong recommendation
Diagnostic suspect	Children with chronic ataxia who have clinical characteristics or markers suggestive of a specific condition, should undergo the analysis of a specific gene or set of genes associated to the observed characteristics.	Strong recommendation
Genotype-phenotype correlation	In children who are positive to mutations associated to Joubert syndrome (JS), Friedreich ataxia (FA) or ataxia telangiectasia (AT), the characterization of the specific gene should be performed for JS, the size of the GAA expansion of the FXN gene should be investigated for FDRA, and the type of mutation of the ATM gene should be defined for AT, to predict the severity and progression of the disease and the possible presence of associated symptoms.	Weak recommendation
FXN gene analysis	In children with chronic ataxia who have no clinical characteristics nor clinical, neuroradiological or biochemical signs suggestive of a specific condition, the analysis of the FXN gene should be performed as a first-choice genetic test.	Strong recommendation
Ataxia-related genes panel	Children with chronic ataxia who are negative to the targeted genetic testing or to the analysis of the FXN gene, should be further assessed by performing the analysis of a panel of ataxia-related genes, even though the accuracy of the test depends on the number and type of genes included in the different panels adopted by each specialized center.	Strong recommendation

^*^ International guidelines indicate as contraindications to lumbar puncture (LP) the presence of signs suggesting increased intracranial pressure, including altered or fluctuating state of consciousness (Coma Glasgow scale <9), relative bradycardia and hypertension, focal neurological signs, abnormal posture or posturing, anisocoric, dilated or non-responsive pupils, papilledema, abnormal “doll's eye” movements. In presence of such signs and symptoms, a neuroimaging exam (e.g., CT) should be performed before considering LP. LP is also contraindicated in case of shock, extensive or spreading purpura, after convulsions until stabilized, coagulation abnormalities, platelet count <100 × 109/liter, anticoagulant therapy, local superficial infection at the LP site, respiratory insufficiency, and severe immune deficiency.

**Table 2:** List of the research recommendations included in the guideline classified according to clinical questions.

Biochemical examinations	In children with Friedreich ataxia, observing lower levels of frataxin appears to be a useful indicator in confirming the diagnosis and monitoring the efficacy of potentially available treatments, but further studies are needed to confirm their utility (including type of tissue and cut-offs). In children with Friedreich ataxia, measuring le levels of cardiac troponin seems to be useful in monitoring cardiac complications, but further studies are needed to confirm its utility.
Genetic testing	Considering the wide heterogeneity of the type of genes included in the various panels adopted in specialized centers, further studies are needed to investigate the accuracy (i.e., sensitivity and specificity) of the different panels, with the objective of identifying a panel analyzing a minimum number of genes associated to the most frequent mutations to be adopted in a homogeneous way. Children with ataxia who are negative to the targeted gene testing, to the analysis of the FXN gene, and to the analysis of a panel of ataxia-related genes, can be further assessed by performing a Whole Exome Sequencing (WES) only for research purposes.
Pharmacological treatments	In children with ataxia telangiectasia the treatment with glucocorticoids, and in particular with intra erythrocyte dexamethasone, seems to be promising in reducing ataxic signs and symptoms, but further large high-quality studies are needed aimed at confirming the efficacy and investigating the safety of the various drugs. Further large high-quality studies are needed to assess the efficacy and safety of administering supplementation with antioxidants for the treatment of motor signs and symptoms in children with a diagnosis of chronic ataxia who do not have any mutation responsible for a deficit of vitamin E or CoQ10.
Care models	Further large high-quality studies are needed to identify and validate indicators of tools to assess the outcomes of the implementation of different care models, to define the most effective and efficient model for the management of children with chronic ataxias and their families/carers.

The full guideline, along with its supplementary material, is available on the Italian National Guideline System website (https://snlg.iss.it/wp-content/uploads/2021/11/LG-Atassie-pediatriche_10-2021.pdf) and on the website of the Center for Rare Diseases of the Italian National Institute of Health (https://www.iss.it/malattie-rare-atassie-linee-guida).

**Figure 1:**
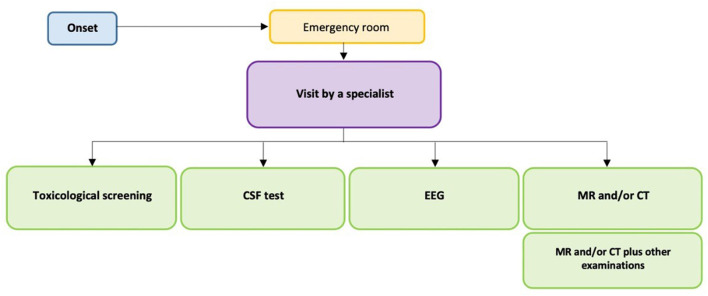
Care pathway for the diagnosis and management of children with acute ataxias.

**Figure 2:**
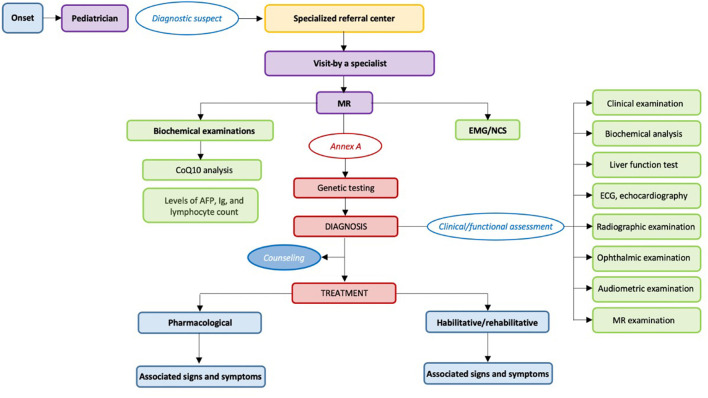
Care pathway for the diagnosis and management of children with chronic ataxias.

The recommendations of this GL were provided by area and not specific to each question, with the aim of making the consequentiality of the management process of the clinical conditions considered as evident and clear as possible, using the graphical representation of the care pathway (Figures 1, 2).

### Acute ataxias

Ataxia in children can be the manifestation of either a chronic or an acute condition. In the case of acute conditions, it can be the consequence of non-fatal acute conditions, but also of conditions that could be life-threatening for the child. It is therefore essential, in the initial diagnostic phase, and particularly in the emergency setting, to identify the cause of ataxia in the timeliest and most appropriate way. All the studies included and analyzed in the GL consistently indicate that the diagnosis should be performed by a specialist (e.g., pediatrician, pediatric neurologist, and emergency physician with training in the management of pediatric, particularly neurological, emergencies). The differential diagnosis is essential to define the diagnostic procedures to be carried out. Patients should be first stabilized (ERC Guidelines for resuscitation 2015) and all conditions requiring immediate urgent care should be treated. After stabilization, the main conditions to be considered are acute post-infectious cerebellar ataxia, which have a prevalence of 36–76% among children referred for acute ataxia to emergency departments, and acute ataxia due to intoxication, which has a prevalence of 8–16%. In the remaining 50% of cases, a diagnostic work-up should be carried out, including MR and other examinations to exclude potentially severe causes.

Generally, post-infectious ataxias cause signs and symptoms lasting <24 h and are more frequent in children aged 1–4 years. In case of suspected intoxication or in case the anamnesis and the objective examination are suggestive of a substance/drug intoxication, performing a toxicological screening test can help to identify the responsible agent, and thus to timely define a therapeutic strategy that can sometimes be lifesaving. The sensitivity of the screening test depends on the number and type of tests included within the standard panel of tests adopted by each referral structure (no data on the specific exams performed were provided by included studies). The appropriate anamnesis and panel of tests should be defined based on the age of the child (e.g., children aged <12/13 years are more likely to have accidentally ingested a substance/drug that was prescribed to them or to a family member, while in adolescents the ingestion of substances/drugs can be voluntary, and an overdose can also happen).

Here, we report the main recommendations for the diagnosis and management of children with acute ataxias, as described in Figure 1 and fully reported in Table 1A.

First of all, the collected and analyzed studies indicate that children with signs of acute ataxia should be referred to or transferred to a specialized pediatric structure only after being stabilized (18). Children referred to an ER for acute ataxia should undergo an accurate anamnesis and a general and specialized objective examination aimed at identifying the possible causes of the signs and symptoms, and at choosing of the type and order of instrumental examination to be carried out.

Regarding diagnostic tests, the main recommendations of LGs are in the following issues:

#### Toxicological screening

Children with signs of acute ataxia whose anamnesis and/or test results do not univocally suggest a defined etiology should be further assessed by performing toxicological screening (e.g., alcohol, amphetamines, cannabinoids, benzodiazepines, ecstasy, methadone, cocaine, and opioids).

#### CSF test

Children with acute ataxia and a suspected infection of the central nervous system (e.g., meningitis, encephalitis) should undergo CSF examination if having no contraindications to lumbar puncture (19).

#### MR and/or CT

Children showing persistent or isolated signs of acute ataxia lasting for more than 3 days, children showing focal signs, visible asymmetric ataxic signs, altered state of consciousness, cranial neuropathy, papilledema and ophthalmoplegia, children with a suspected demyelinating or vascular disease, and children with an anamnesis of head trauma should undergo brain CT and/or MR.

### Chronic ataxias

Here, we report the main recommendations for the diagnosis and management of children with chronic ataxias, as described in Figure 2 and Table 1B.

#### Diagnosis

In case of suggestive signs and symptoms or a suspected diagnosis, pediatricians and/or specialists on the territory should refer the child to a specialized referral center. This is because the diagnostic process includes specific tests, such as MR and genetic testing (20), which should be performed by specialists and require to be included within a counseling framework. Chronic ataxias are included within the definition of complex diseases. This type of disease requires a care model that takes into consideration the need of these children for complex management provided by several specialized physicians and healthcare professionals. Unfortunately, chronic ataxias are also rare diseases, thus the viability of creating permanent specialized multidisciplinary teams, for stroke units, is extremely low, while building networks of specialized professionals providing coordinated healthcare appears to be the most feasible solution. These networks could identify the SRC as the access point coordinating the management by referring children to the networks of specialized professionals with whom it is constantly in communication. Each SRC should include a minimum set of specialized physicians (e.g., cardiologist, geneticist, physiatrist, neurologist, child neuropsychiatrist, orthopedist, pediatrician, pneumologist, and psychologist) and healthcare professionals (professional health educator, physiotherapist, nurse, speech and language therapist, orthoptist, psychiatric rehabilitation therapist, orthopedic therapist, child neuro-psychomotor therapist, occupational therapist, podiatrist, and psychologist/neuropsychologist) among its staff. In case of the absence of a specific professional, it should refer the patient to external specialists.

Regarding diagnostic tests, the main recommendations of LGs are in the following issues:

##### MR imaging

Considering how essential a timely (and early when possible) diagnosis is for patients/family members and clinicians, it is crucial to reach it in the most effective way in terms of both time and resources. Most of the time the definite diagnosis is reached through genetic testing, therefore choosing in the most efficient way the type and sequence of tests to be performed is essential, and MR imaging can be useful, along with clinical examination, in orienting this choice. Performing MR imaging in an early phase of the diagnostic process is, in fact, useful to identify structural signs such as atrophy or to identify potential malformations or ischemic and metabolic conditions. Specific MR patters can be diagnostic or suggestive of a type or a category of ataxias, thus guiding the choice of the subsequent diagnostic strategy. As an example, literature univocally indicates that RM results reporting the presence of the so-called molar tooth sign (MTS) are diagnostic of Joubert Syndrome (JS).

##### Biochemical examinations

Considering that some ataxic signs and symptoms can be due to potentially treatable causes, such as metabolic conditions and vitamin or enzyme deficits, excluding causes such as metabolic deficits can be useful as a first-line approach. Gathered evidence supports that observing low levels of CoQ10 in the muscle tissues of children with a diagnosis of chronic ataxia can be suggestive of potentially treatable causes of ataxia due to a deficit of CoQ10. Moreover, several chronic ataxias are associated with specific biochemical patterns that might be suggestive or supportive of a diagnosis. Literature showed that high levels of AFP in children with suspected chronic ataxia, along with a profile of low levels of IgA, IgE, and IgG and a low lymphocyte count (CD4+ and CD8+), is suggestive of and supports an early diagnosis of Ataxia telangiectasia.

##### Genetic testing

**Figure 3:**
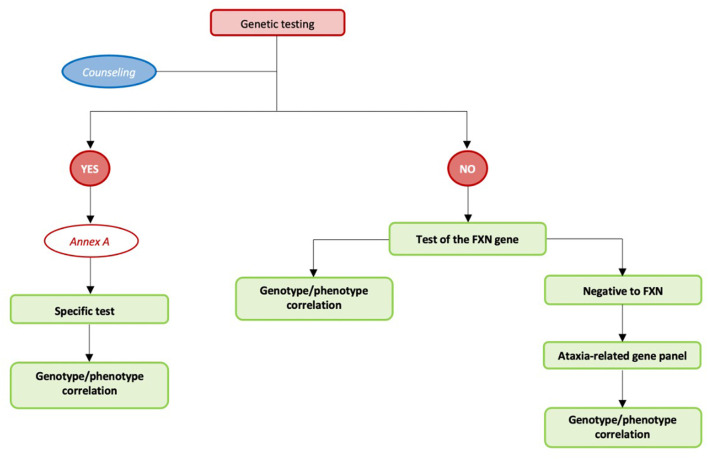
Care pathway for diagnostic testing children with chronic ataxias.

Reaching a definite diagnosis is a primary objective for both patients/family members and clinicians to allow for planning the best available care option. Therefore, it is essential to reach this diagnosis in the most efficient way in terms of time and resources. A national law instituted a rare diseases network to develop preventive and surveillance actions, improve interventions for diagnosis and treatment, and support information and training programs. The network includes all regional services that have specific expertise in the diagnosis and management of rare diseases, along with adequate equipment and complementary services (e.g., biochemical or genetic diagnosis) (Decreto ministeriale n. 279 del 18 maggio 2001) (Ministero della salute. Rete nazionale malattie rare. http://www.salute.gov.it/portale/temi/p2_6.jsp?-lingua$=$italiano&id$=$707&area$=$Malattie%20rare&menu$=$-vuoto). For this reason, identifying any clinical, neuroradiological, and biochemical sign suggesting the use of a specific genetic test is extremely important to reach a definite diagnosis in the shortest possible time and reducing the ambiguities/difficulties in interpreting genetic test results. In absence of specific signs suggesting a suspected diagnosis, the most efficient and effective strategy is to consider testing for the most frequent conditions. In case testing for a specific test or testing for Friedreich Ataxia (the most frequent chronic ataxia in the Italian population) did not result in a positive result, a molecular test should be performed using a panel of genes that have currently been proven to be associated with ataxia. If all the above-mentioned genetic tests did not produce any positive results, a whole exome sequencing (WES) approach can be considered (Table 1 and Figure 3).

#### Clinical/functional assessment of non-ataxic signs

Pediatric ataxias include a group of heterogeneous conditions causing the main ataxic signs, and a variety of other non-ataxic signs and symptoms, including complications involving specific organs (e.g., heart and liver) and physical deformities or malformations.

The presence of sometimes progressive associated conditions (e.g., hearing and/or visual impairment), including malformations or deformities (e.g., scoliosis and pes cavus) can determine varying degrees of disability associated with stress, discomfort, and sometimes pain. Appropriate management and, where possible, the treatment of malformations and deformities can significantly reduce their overall burden and often decrease, or even prevent, consequences, such as stress, pain, and discomfort. The early treatment, when possible, can solve or help improving motor and non-motor symptoms in children, while, in absence of a disease-modifying treatment, an appropriate management of the condition can be essential in improving their quality of life (QoL) and the QoL of their families/caregivers. Therefore, all children with chronic ataxia should undergo an adequate clinical assessment aimed at identifying and characterizing any potential malformation/deformity (e.g., scoliosis and pes cavus/planus) or any other non-ataxic clinical condition (e.g., visual/auditory impairment and conditions affecting organ function) to define the most appropriate intervention strategies.

##### Visual and audiometric examinations

The presence of hearing and visual defects can cause discomfort and stress, but also different other cognitive, learning, and relational consequences. Both tests are non-invasive and relatively easy-to-perform procedures. Evidence indicates that children with chronic ataxia should undergo an ophthalmic and visual examination aimed at identifying possible neuro-ophthalmic and visual symptoms. Moreover, the literature suggests that children with Friedreich ataxia, in particular, should undergo a clinical and audiometric examination in an early stage of the diagnostic process.

##### Echocardiographic exam

Early-onset Friedreich ataxia of often associated with Hypertrophic Cardiomyopathy) that, being sometimes asymptomatic, might remain undiagnosed. Therefore, children with FA, even if having no signs of cardiomyopathy, should undergo a clinical, electrocardiographic, and echocardiographic exam aimed at identifying possible cardiac anomalies (e.g., hypertrophic cardiomyopathy) and, in case, assessing their severity and progression.

##### Radiographic examination

As some types of chronic ataxia may be associated with the presence of spinal deformities, such as scoliosis, children with chronic ataxia should undergo a specific radiographic examination aimed at identifying the presence of scoliosis and, in case, assess its progression. However, as ataxia telangiectasia is characterized by a higher susceptibility to infections, a higher sensitivity to radiations, and a higher risk of cancer, it is necessary to limit as far as possible the number of RX exams in this population, to minimize the exposure to radiation and the subsequent neoplastic risk.

##### Biochemical examinations

As some ataxias can be associated with several complications involving organ function or the immune response, monitoring some biochemical parameters can be useful to prevent or treat such conditions. AT is usually associated with immune disorders, therefore routine immunological tests for Ig and lymphocyte count can be predictive of such disorders. Gathered literature suggested that monitoring Ig levels (IgA, IgE, IgG, and IgM) and lymphocyte count in children with AT can be useful to characterize an immunodeficiency profile and therefore prevent the risk of infections. Moreover, strong evidence indicated that observing high levels of IgM in these children is suggestive of a phenotype characterized by a higher risk of infection and earlier mortality. Moreover, as AT can be also associated, in later stages, with a form of hepatopathy, children with ataxia telangiectasia, starting from their tenth year of age, could benefit from a liver function test.

#### Counseling

The rarity of chronic pediatric ataxias, along with the heterogeneity of their clinical presentation, inevitably causes family members/caregivers to face their child's condition and manage it through healthcare services from obtaining a diagnosis to granting their child the appropriate management. The difficulties raising from all the steps needed to manage a child with a rare disease determine a burden for their family members/caregivers that can cause them significant physical and psychological health risks.

The only sources of support that are currently available for parents/caregivers to provide them indications on the healthcare structures that they can contact or to share their practical and emotional experiences are the associations and self-help groups. However, the responsibility of helping parents/caregivers navigate through the healthcare system and thus coordinating the child's care cannot and must not be demanded exclusively by the associations. Associations can have the role of helping families/caregivers in identifying the access point to healthcare, but once within services, a definite care path should be available, along with a coordinated network of specialized professionals involved in the care plan.

Another extremely relevant aspect is the impact that the communication of the diagnosis has on the children and their families/caregivers, and the consequence that this diagnosis has in terms of both their psychological and emotional reactions. Pediatric ataxias can cause signs and symptoms with a widely variable grade of severity and can determine extremely heterogeneous degrees of disability and prognostic trajectories. In some cases, accepting and elaborating a diagnosis of a rare disease can have relevant consequences, and can turn into an extremely complex and painful process for both children and their families/caregivers. Therefore, it is essential to support them by offering them targeted and tailored psychotherapeutic and rehabilitative interventions (e.g., psychoeducational or behavioral activities) and/or rehabilitative or recreational interventions involving both children and their families/caregivers (e.g., sensorial activities).

#### Care plan

The specialized referral center (SRC), after reaching a definite diagnosis, should define a care plan. The plan should then be applied by the territorial unit closer to each patient's residence while maintaining a constant coordination with the SRC.

#### Treatment

##### Pharmacological interventions

No pharmacological treatments for chronic ataxia are currently supported by available evidence. In children with Friedreich ataxia, currently available evidence does not support the treatment with idebenone or interferon gamma-1b, as they appear to be not effective in reducing ataxic signs. When considering potentially treatable ataxias, no evidence currently supports treatment with vitamin E or CoQ10 supplements in children with a genetic mutation responsible for a deficit of these substances. However, considering that signs and symptoms in these ataxias are due to the deficit caused by the mutation, supplementation with vitamin E or CoQ10 should be initiated as soon as possible.

##### Habilitative/rehabilitative

The motor and cognitive habilitative/rehabilitative treatment remains the first-line therapeutic approach for these conditions. Their rarity and the variety and heterogeneity of the sign and symptoms that they include makes it essential to define personalized rehabilitative plans, tailored to the children's needs and expectations, thus virtually impossible to standardize and generalize. The motor and cognitive rehabilitative strategy should be included in a rehabilitative project shared with the children, their families/caregivers, and the healthcare professionals who participate in their management. The rehabilitative project should start by performing an assessment of the abilities, competences, and occupational performances of each child, the type and severity of their signs and symptoms, and their functional prognosis, where assessable. This assessment allows to target the interventions to the expectations, needs, and actual abilities of each child, and to establish the synergistic strategies for the intervention and the coordinated action of the healthcare professionals. Rehabilitation should be focused on the treatment of children with different levels of signs and symptoms, disabilities, and/or dysautonomia, and should be also provided to children who are autonomous and have mild symptoms to prevent worsening and avoid the possible onset of new symptoms. The initial assessment and the subsequent evaluations aimed at determining the effects of the motor and cognitive rehabilitative strategy should be carried out using appropriate tools that have been validated in a population of children with pediatric ataxia. Therefore, despite the availability of widely adopted tools, further studies are needed to support the validity and reliability of different tools specifically for the assessment of children with pediatric ataxia.

##### Treatments of non-ataxic signs

The therapeutic strategies defined within the rehabilitative project should target both the treatment of the motor signs and symptoms and the non-motor symptoms associated with the different forms of ataxia, including the use of aids and/or orthoses where necessary. As an example, though orthopedic corsets and shoes are used as the first-line approach for the management of scoliosis and foot deformities (e.g., pes cavus or pes planus), they might not be appropriate if they restrict the child's mobility or gait or cause other manifestations limiting their tolerability (e.g., pain and discomfort).

## Discussion

The Guideline Development Working Group, despite the lack of evidence or the methodological limitations of the available evidence, is deemed essential to provide indications and recommendations, in particular, in some clinically relevant areas. Research recommendations were also defined, where indicated, to provide directions and support to future research in this field. The WG adopted and supported a more pragmatic approach in producing the guideline, with the objective of designing a document that could be actually useful and implementable in clinical practice, including also recommendations for research (Table 3). For this purpose, recommendations were classified in areas, to highlight and follow as clearly as possible the sequence of the steps required within the care process, which were also summarized and visually represented in the care pathway. Considering the complexity of these rare diseases, the WG deemed it essential to underline through the care pathway and throughout the whole structure of the guideline the unequivocal need to adopt integrated and complex approaches for the diagnosis and management of children with ataxias, considering these conditions in a holistic way.

**Table 3:** Annex A of the guideline. Main characteristics of the most frequent pediatric chronic ataxias.

**Diagnosis**	**Characteristics**				
	**Onset**	**Neurological signs and symptoms**	**Multisystem signs and symptoms**	**Neuroimaging**	**Genes**
Friedrich ataxia	< 25 years	Swallowing disorders, progressive gait ataxia, dysarthria, peripheral axonal neuropathy with absence of deep tendon reflexes	Scoliosis, pes cavus, non-obstructive hypertrophic cardiomyopathy, urinary dysfunction, diabetes mellitus, deafness	Generally normal MR with possible reduced cerebellar peduncle volume	FXN
Ataxia telangiectasia	1–4 years	Progressive cerebellar ataxia, oculomotor apraxia, dysarthria, peripheral axonal neuropathy, absence of deep tendon reflexes	Telangiectasia, non-progressive immunodeficiency, lung and nasal cavities diseases,	MR with signs of cerebellar atrophy	ATM
Ataxia with oculomotor apraxia type 1	2–10 years	Cerebellar ataxia, horizontal and vertical oculomotor apraxia, ophthalmoparesis, peripheral axonal neuropathy with absence of deep tendon reflexes, chorea, upper limb dystonia	Pes cavus, generally atrophic hands and feet	MR with signs of cerebellar atrophy	APTX
Ataxia with oculomotor apraxia type 2	3–30 years	Cerebellar ataxia, mild/moderate oculomotor apraxia, peripheral axonal neuropathy with absent or reduced deep tendon reflexes	Rare telangiectasia	MR with signs of cerebellar atrophy (including cerebellar hemispheres and vermis)	SETX
Ataxia with vitamin E deficiency	5–15 years	Slowly progressing ataxia, loss of hand coordination and proprioception, absence of deep tendon reflexes, dysdiadochokinesia, dysarthria	Decline of visual acuity	MR with signs of cerebellar atrophy (50% of cases)	TTPA
Autosomal recessive spastic ataxia of Charlevoix Saguenay	12–18 months	Progressive cerebellar ataxia, nystagmus and dysarthria, demyelinating peripheral neuropathy with loss of deep tendon reflexes	Lower limb spasticity, foot deformities	MR with evidence of vermis atrophy with superior predominance and/or cerebellar hemispheres atrophy and linear pons hypo intensity	SACS
Joubert syndrome	Birth/early life	Hypotonia, nystagmus, oculomotor apraxia, ataxia, developmental delay and intellectual disability, early life tachypnea and/or apnea	Retinal, ocular, renal, liver, craniofacial, digital and skeletal disorders	MR with molar tooth sign (MTS)	>40

Main characteristics of the most frequent pediatric chronic ataxias.

Considering the crucial importance of providing clear indications for the management of pediatric ataxias, this GL was developed taking into consideration throughout the whole process the context of each produced recommendation, and their feasibility in clinical practice. For this purpose, two specific review questions were included within the guideline, aimed at identifying the most effective EBM care models to be adopted for pediatric ataxias. In the evidence review process for these questions, the WG analyzed all potential facilitating factors and obstacles to the implementation of the care model that was being recommended by the GL and proposed within the care pathway. The care pathway was produced as a tool to facilitate the dissemination and implementation of the contents and recommendations produced by the GL. The interactive version of the pathway is available on the Italian National Guideline System website along with a leaflet dedicated to families and caregivers of children with ataxias.

The care pathway is aimed at facilitating the contextualization and implementation of the recommendations, and its objective is to provide a methodology to organize the management and care process of a defined group of people in a specific period. The care pathway elaborated for this guideline included all the key elements that are essential to provide EBM care. Therefore, it took into consideration the values and preferences of patients and their families/caregivers, key facilitators for the communication and interaction among healthcare professionals and families/caregivers, facilitators for the coordination of the diagnostic and care process, defining the roles and sequence of activities along with the appropriate resources required for each step.

## Data availability statement

Datasets are available on request: The raw data supporting the conclusions of this article will be made available by the authors, without undue reservation.

## Ataxia guideline development working group (GDWG)

Bacigalupo I, Beccani L, Bellomo G, Bertini E, Biagiotti S, Borgatti R, Bramati A, Cammarano R, Ceccarini A, Corti C, Della Gatta F, Della Seta M, Faccioli S, Gainotti S, Gaudiano A, Gervasi G, Ginevrino M, Mariotti C, Mayer F, Mennini FS, Micalizzi A, Nuovo S, Pandarese D, Parisi P, Penna L, Petrarca M, Pioggia G, Raucci U, Romaniello R, Sacchi D, Salvaggiulo V, Sciattella P, Schirinzi T, Tofani M, Torreri P, Valente EM, Vasco G, Zaccaria V, Zanni G.

## Author contributions

EL and PP: conceptualization, data curation, methodology, visualization, and writing—original draft. LV and LS: methodology and writing—review and editing. DT: conceptualization and writing—review and editing. NV: conceptualization, funding acquisition, supervision, and writing—review and editing. All authors contributed to the article and approved the submitted version.

## Funding

The project was funded by the Italian Ministry of Health: NET-2013-02356160—Pediatric ataxias and Public Health: epidemiological studies and disease registry, characterization of genetic determinants and implementation of protocols for diagnosis, management, and rehabilitation using innovative low cost, widely accessible technologies.

## Conflict of interest

The authors declare that the research was conducted in the absence of any commercial or financial relationships that could be construed as a potential conflict of interest.

## Publisher's note

All claims expressed in this article are solely those of the authors and do not necessarily represent those of their affiliated organizations, or those of the publisher, the editors and the reviewers. Any product that may be evaluated in this article, or claim that may be made by its manufacturer, is not guaranteed or endorsed by the publisher.
